# Optimizing greenhouse microclimate for plant pathology: challenges and cooling solutions for pathogen control in arid regions

**DOI:** 10.3389/fpls.2025.1492760

**Published:** 2025-02-06

**Authors:** Abdulmujib G. Yusuf, Fahad A. Al-Yahya, Amgad A. Saleh, Ahmed M. Abdel-Ghany

**Affiliations:** ^1^ Plant Protection Department, College of Food and Agricultural Sciences, King Saud University, Riyadh, Saudi Arabia; ^2^ Faculty of Energy Engineering, Aswan University, Aswan, Egypt

**Keywords:** greenhouse technology, pathosystems, plant-pathogen interaction, sustainable agriculture, climate change, disease control, arid regions

## Abstract

Crop production using greenhouse technology has become increasingly essential for intensifying agricultural output, particularly in regions with challenging climatic conditions. More so, greenhouses do not only support continuous crop supply but also provide a controlled environment crucial for studying plant-pathogen interaction. Likewise, pests and diseases are a constant threat to crop production, which requires innovative control methods. Providing a suitable and sustainable control method requires a detailed probe into the relationship between plants and biotic disturbance under controlled settings. Therefore this review explores the relationships between plants and pathogens, highlighting the impact of extreme greenhouse microclimates on plant pathology assays. Given the extreme weather conditions in the Arabian peninsula, the efficiency of greenhouses, especially during summer, is compromised without adequate cooling systems. This review discusses the current strategies employed to optimize greenhouse conditions in hot arid regions, aiming to enhance plant health by mitigating pathogen activity while minimizing energy, and water consumption. The review also provides an overview of how microclimatic parameters within greenhouses influence plant-pathogen dynamics, ensuring conditions that are conducive to managing both biotic and abiotic diseases. Additionally, the review aims to evaluate various cooling techniques available and most widely accepted in hot arid regions. Moreover, the performance indicators, principles, and effectiveness of each technique are discussed. Promising advances in the manipulations and combination of these techniques have proven to maintain an appropriate greenhouse microclimate with minimal resource use.

## 1 Introduction

To meet the growing global demand for food while minimizing biodiversity loss, greenhouse technology has emerged as a sustainable and efficient crop production method (Aznar-Sánchez et al., 2020). Generally, greenhouses are employed to provide and regulate environmental conditions that support optimal plant growth in a controllable non-natural environment. Beyond enhancing productivity, greenhouses play a pivotal role in plant science research by enabling precise control of environmental conditions. This is particularly beneficial for investigating host-pathogen interactions, which are often influenced by climatic variables (Lahlali et al., 2024). In controlled settings, greenhouses facilitate the study of disease dynamics, allowing researchers to simulate conditions conducive to specific plant-pathogen interactions that would otherwise be difficult to reproduce in open fields.

Despite these advantages, plant diseases remain a persistent threat to agricultural productivity. More so, the spatial and temporal variability of pathogens (Dai et al., 2022), along with their ability to evolve into strains capable of overcoming host resistance (McLaren and Callahan, 2020), underscores the importance of understanding pathogen dynamics. Greenhouses offer an invaluable platform for such studies, providing the controlled environments necessary to probe the complexities of host-pathogen relationships. Nonetheless, conducting such research in greenhouses located in hot arid regions presents unique challenges, particularly in maintaining optimal conditions for both plants and pathogens.

Primarily, controlled environments are created by managing climatic variables and selecting appropriate covering materials. Despite the simplicity of the setup, the greenhouse presents dynamic processes that require delicate regulations. The controlled greenhouse has been considerably achieved in temperate climates whereas greenhouses in hot arid regions face technological challenges due to extreme weather conditions, especially in summer (Ghoulem et al., 2019). While greenhouses offer controlled environments for plant pathology research, they face significant challenges in hot arid regions, particularly in managing energy and water consumption.

Regions like the Arabian Peninsula, characterized by extreme weather conditions, present significant obstacles to greenhouse operations. High ambient temperatures, coupled with limited freshwater availability, strain conventional cooling systems, which are critical for creating favorable microclimates inside greenhouses. Consequently, without adequate cooling, the efficiency of greenhouses for both crop production and plant pathology research is severely compromised. All the same, the high energy and water demands of cooling systems (Abedrabboh et al., 2024) exacerbate the sustainability challenges in these resource-scarce regions.

Given these constraints, innovative approaches to greenhouse cooling are required to balance resource efficiency with the need for optimal conditions. Even though strategies such as evaporative cooling and the use of advanced materials for greenhouse structures have shown promise (Soussi et al., 2022), there are still obvious limitations, particularly in regions with saline water sources that clog cooling systems. Consequently, the efficiency of these strategies under the intense solar radiation of arid climates remains a critical research area. Taken together, these are necessities that need to be observed in controlling the high temperature inside greenhouses in hot arid regions with the minimal use of resources.

Therefore, the current review intends to bridge this gap, especially cooling the greenhouse for plant science studies. This review examines the interplay between greenhouse microclimates and plant-pathogen dynamics, focusing on the challenges faced in hot arid regions. It explores the current state of greenhouse cooling technologies, evaluates their effectiveness, and highlights opportunities for optimizing resource use. Special attention is given to identifying sustainable solutions tailored to the unique environmental conditions of the Arabian Peninsula. By addressing these gaps, this review aims to contribute to the development of resource-efficient greenhouses capable of supporting both agricultural productivity and advanced plant pathology research.

## 2 Environmental influences on plant diseases

Plants are constantly influenced by biotic and abiotic factors (Chamard et al., 2024), which can either support or hinder their growth and health. The quality and yield of crops are determined by the complex interactions between plants, pathogens, and environmental conditions. Among these, the environment plays a pivotal role in shaping the outcomes of plant-pathogen interactions (Scholthof, 2007). Favorable environmental conditions often exacerbate pathogen activity (Lahlali et al., 2024), leading to devastating impacts on agriculture, significant economic losses, and threats to food security.

Diseases are manifested as symptoms in plants (Özkaya et al., 2024), signifying disturbance of their normal physiological functions. There is a need to curb the excesses of plant diseases on plants which can be achieved through a thorough knowledge of the causal pathogens, and how their activities bring about the disease situation. To control pathogens effectively, a comprehensive understanding of their disease-inducing activities is essential. Similarly, it’s important to understand their epidemiologic potential ensuing losses, which are relevant to mitigatory options. This knowledge forms the foundation of plant pathology, a field that has evolved from classical diagnostic methods to the incorporation of advanced tools like gene cloning, genetic engineering, and software-assisted modeling. These advancements enable precise disease management in both open-field and controlled environments, including greenhouses (Vidhyasekaran, 2004; Mojerlou, 2024). In the open field, disease incidence and density are determined by the host, the pathogen, and the environment (Mojerlou, 2024). Typically, a virulent pathogen interacting with a susceptible host in a conducive environment leads to disease. Understanding the dynamics of this interaction is essential for predicting outbreaks and implementing timely interventions. Furthermore, environmental factors such as temperature, humidity, and light are not only critical for disease progression but also for plant growth and productivity (Lahlali et al., 2024). Environmental conditions significantly impact disease progression and plant growth (Singh et al., 2020), necessitating innovative strategies like shielded cultivation to protect plants from biotic and abiotic stresses, ensuring optimal growth conditions. Generally, innovative strategies, such as protected cultivation systems, are increasingly used to mitigate the adverse impacts of biotic and abiotic stresses. Primarily, greenhouses, for instance, offer a controlled environment to optimize growth conditions while reducing exposure to unfavorable external factors. Altogether, the advancement provides a unique opportunity to study and manage plant-pathogen interactions, ensuring sustainable crop production even in regions with extreme climates. Thus, integrating environmental insights with advanced tools and cultivation techniques is vital for safeguarding plant health and productivity in the face of evolving agricultural challenges.

## 3 The role of protected cultivation in plant pathology research

Protected cultivation has long been a cornerstone of agricultural innovation, offering a controlled environment to mitigate the effects of adverse weather conditions. Traditionally, farmers employed simple methods such as growing annual crops under the shade of perennial trees to regulate temperature and humidity during their tender stage (Beer et al., 1997). For example, a cultural practice among the cocoa farmers of southwestern Nigeria is spreading cocoa seeds under the shade of banana plants till seedling emergence after which the cocoa seedlings will be transplanted onto the open field (Famuwagun et al., 2018). This is done to prevent the seedling from environmental stress associated with direct sowing on the field. Similarly, elaborate bamboo stakes and palm fronds were used as shade structures for high-value crops, with shade levels tailored to the crop’s growth stage. In the nursery, about 70% shade level is required for grooming young cocoa plants while about 40% is required for mature cocoa plants (Asare and David, 2010). This is because the photosynthetic mechanisms of the leaves and flowers of cocoa plants are damaged when exposed to a high temperature above 30 °C (Famuwagun et al., 2018). This highlights the ingenuity of early agricultural systems in adapting to environmental challenges. Modern protected cultivation systems, such as greenhouses and screenhouses, have revolutionized agriculture by providing an optimal environment for plant growth and research. These systems not only enhance productivity and resource efficiency but also facilitate advanced studies in plant pathology by enabling precise control over environmental conditions.

### 3.1 Greenhouses: a controlled environment for pathology assays

Greenhouses represent one of the most advanced forms of protected cultivation (Hanan, 2017), offering a controlled setting for crop production and scientific research. They are indispensable in plant pathology research, providing an environment where plant-pathogen interactions can be meticulously studied. By isolating variables such as temperature, humidity, and light, researchers can investigate the effects of environmental factors on disease development and test mitigation strategies under controlled conditions.

Greenhouse technology is also critical for addressing global food security challenges (Khan et al., 2021). With the global population projected to reach 9.7 billion by 2050, agricultural production must increase by 70% to meet food demand (FAO, 2009). To meet this required estimated increment in world food production, the current amount of arable land needs to be extended or face an overbearing intensification (Alexander et al., 2015). However, the intensification of available arable land for agricultural purposes will require more input, such as agrochemicals, which pose additional environmental, human, and health-related hazards (Tilman, 1999) with a sizable amount of disturbances to many terrestrial and aquatic ecosystems. Taken together, we will agree that expanding arable land or intensifying agricultural practices often comes at the expense of biodiversity and ecosystem health (Aznar-Sánchez et al., 2019). Nonetheless, in as much as meeting the projected increase in food demand is important, a vital challenge for sustainable natural resources management is finding a balance between production and preservation of the natural ecosystem. Consequently, there is a dire need for greenhouse technology to mitigate this challenge. In achieving this goal, agricultural practices should shift from intensive to non-intensive through the adoption of improvement in agricultural technologies, minimal use of agrochemicals, and promotion of protected cultivation.

Greenhouses provide a sustainable alternative by optimizing resource use, reducing reliance on agrochemicals, minimizing environmental impact (Aznar-Sánchez et al., 2020),. In addition, greenhouses allow for off-season crop production, enabling tropical crops to be cultivated in temperate regions or arid climates where traditional farming methods are less viable (Lefers et al., 2020). However, despite their advantages, greenhouses in hot arid regions face significant challenges. Extreme temperatures, particularly during summer, demand substantial energy inputs for cooling (Bicer et al., 2022), complicating their sustainability. Addressing these challenges requires innovative cooling systems and efficient resource management to balance productivity with environmental conservation.

### 3.2 Screen houses and plastic net houses: alternatives and applications

During summer, the main factor limiting protected cultivation in arid regions is the overheating of air in greenhouses. The daily outdoor average ambient air temperature often exceeds 45°C in the period from late April to late September. The greenhouse effect increases the air temperature in the uncooled greenhouse reaching over 60°C (Abdel-Ghany et al., 2015). Consequently, an efficient greenhouse cooling system is required to maintain plant growth-friendly temperatures. With natural ventilation, around 100 kJ of accumulated heat per m^2^ of floor area needs to be removed from the greenhouse every day (Abdel-Ghany et al., 2016). Thus, in regions with high ambient temperatures, such as the Arabian Peninsula, screen houses and plastic net houses offer an alternative to traditional greenhouses. Net houses offer several advantages, including reduced transpiration, lower pesticide use, and protection against wind, pests, and excessive solar radiation (Sotelo-Cardona et al., 2021).

These structures provide partial protection against environmental stressors while reducing the energy and water demands associated with greenhouse cooling. For instance, net houses have been shown to reduce water consumption by up to 13 kg m² day^-^¹ in summer and energy use by 0.26 kWh m² day^-^¹ compared to traditional greenhouses (Abdel-Ghany et al., 2016). However, their effectiveness is limited in extremely hot climates. While they mitigate some heat stress, they lack the full environmental control of greenhouses, resulting in higher daytime temperatures and lower photosynthetically active radiation (PAR) transmission compared to cooled greenhouses (Soussi et al., 2022; Alhelal et al., 2024). Consequently, while net houses are suitable for certain crops and conditions, greenhouses remain essential for cultivating high-value crops and conducting pathology research in hot arid regions.

Therefore, the net house serves as a form of complementary system to further enhance resource efficiency, earmarking them as a valuable unit in productive agricultural systems.

## 4 Greenhouse microclimatic parameters and their management

Crop performance requires optimal microclimatic parameters which should be catered for by the greenhouse control system, especially in arid regions where the outside weather parameters are at the extreme. Greenhouse cultivation is a popular adventure in the arid region with about 1300 ha of protected crops in Gulf countries alone (FAO, 2021). Greenhouses in this region not only enable water-efficient agriculture but also enhance pest and disease control, with productivity levels up to five times greater than open-field systems (Ghoulem et al., 2019). These attributes show the significance of greenhouse cultivation to the weather-disadvantaged Arabian Peninsula. However, these advantages come with high energy and water demands (Gorjian et al., 2021), especially during the summer months where energy expenditure presents the largest impact on the greenhouse systems and the environment (Ahemd et al., 2016). Addressing these challenges, thus, requires integrating climate-smart agriculture with advanced greenhouse management strategies. This will mitigate crop exposure to harsh internal conditions like heat scorching and the risk of a high disease incidence due to high relative humidity (RH).

### 4.1 Radiation and temperature regulation in greenhouses

Greenhouse temperature especially during the daytime builds up from the amount of solar radiation that is allowed into the greenhouse (Lamnatou and Chemisana, 2013; Colantoni et al., 2018). The temperature accrued is subsequently stored in mediums within the greenhouse and later serves as a source of heat during the nighttime (Papasolomontos et al., 2013). Daytime temperatures in a greenhouse are largely influenced by solar radiation, with the spectrum between 400 and 700 nm which is referred to as photosynthetic active radiation (PAR), being used for photosynthesis (Wells, 1997). The plant absorbs most of the PAR for use in its photosynthetic process, and heat is generated from the radiation balance. The PAR can be influenced by a variety of factors, including the characteristics of the specific greenhouse (Colantoni et al., 2018). For instance, the amount of radiation transmitted and available at the crop level in the greenhouse is influenced by the greenhouse covering material and incident irradiation. Also, a sufficient amount of light is required in the greenhouse for optimal plant performance (Paradiso and Proietti, 2022). However, in as much as light enhances photosynthesis and overall plant growth, its extreme can also negatively affect plant performance because its excess can cause heat stress. Particularly, in young plants, whose high rate of evaporation makes them vulnerable to scorching (Soussi et al., 2022). In essence, the greenhouse, particularly the design, plays a crucial role in temperature management. Studies have shown that actors such as covering materials, window placement, and orientation must optimize light transmission while minimizing heat buildup (Paradiso and Proietti, 2022; Zhang et al., 2022). For most crops, the optimal temperature range is 10–24°C (Subahi and Bouazza, 2020). Beyond this range, artificial cooling systems become necessary to prevent heat stress, which can weaken plants and increase susceptibility to pests and disease (Huot et al., 2017). For example, the root disease of clover has been shown to escalate under adverse soil temperatures (You et al., 2017). Despite the importance of temperature, disease outcome is however affected by combinations of different environmental factors including available moisture.

### 4.2 Humidity and gaseous exchange (H_2_O and CO_2_)

A very important parameter to be considered in maintaining healthy plant conditions in the greenhouse is the amount of water vapor therein otherwise referred to as relative humidity. Water stress is induced in crops at lower RH, and this low level can lead to a reduction in shoot development (Soussi et al., 2022). However, the reactions of crops to RH vary. It is observed through different Various studies have shown that low humidity can enhance plant quality in certain crops, while triggering opposite effects in some other crops (Mortensen, 2000). For example, the leaf area of cucumber has been observed to increase at high humidity while that of tomato decreases Nevertheless, maintaining an optimal RH level (45-85%) is critical for water regulation, leaf development, and tissue elongation (Fanourakis et al., 2020; Chowdhury et al., 2021).

High humidity can also be a factor in crop disease incidence in the greenhouse (Kruidhof and Elmer, 2020), thus affecting the quality of yield. High humidity can promote fungal diseases, such as *Botrytis cinerea*, by facilitating spore germination on leaf surfaces, which can be managed by lowering the high humidity in the greenhouse (Yunis et al., 1990). Controlled ventilation systems are key to managing RH, especially during the nighttime when restricted air exchange often leads to condensation and high humidity levels (Amani et al., 2020). However, certain situations, such as calcium pressurization or biocontrol application, may require elevated RH levels. Therefore, greenhouse systems must be adaptable to specific crop needs and environmental conditions.

In addition to water vapor, carbon dioxide (CO_2_) levels are a critical greenhouse parameter. Elevated concentration, driven by climate change, can initially enhance photosynthetic rates and plant biomass (Cooper et al., 2002). However, prolonged exposure, in the long run, can result in the downregulation of the net photosynthetic (Kirschbaum, 2011). Notably, elevated CO_2_ can also influence plant-pathogen interactions.

Biotrophic pathogens benefit from increased carbohydrate levels in the host tissue, leading to larger canopy size, and more biomass (Smith and Luna, 2023). However, elevated CO_2_ levels have been shown to accelerate disease progression in some crops while leaving others unaffected, such as pathogens adapted to dry soils (Hibberd et al., 1996; Mieslerová et al., 2022).

## 5 Interactions between microclimatic factors and plant-pathogen dynamics

### 5.1 Influence of temperature on plant-pathogen relationships

The direct effect of the microclimatic parameters may bring about changes in plants and/or the plant community, and the changes in the plant formation may affect infection (Burdon, 1987).

**Figure 1:**
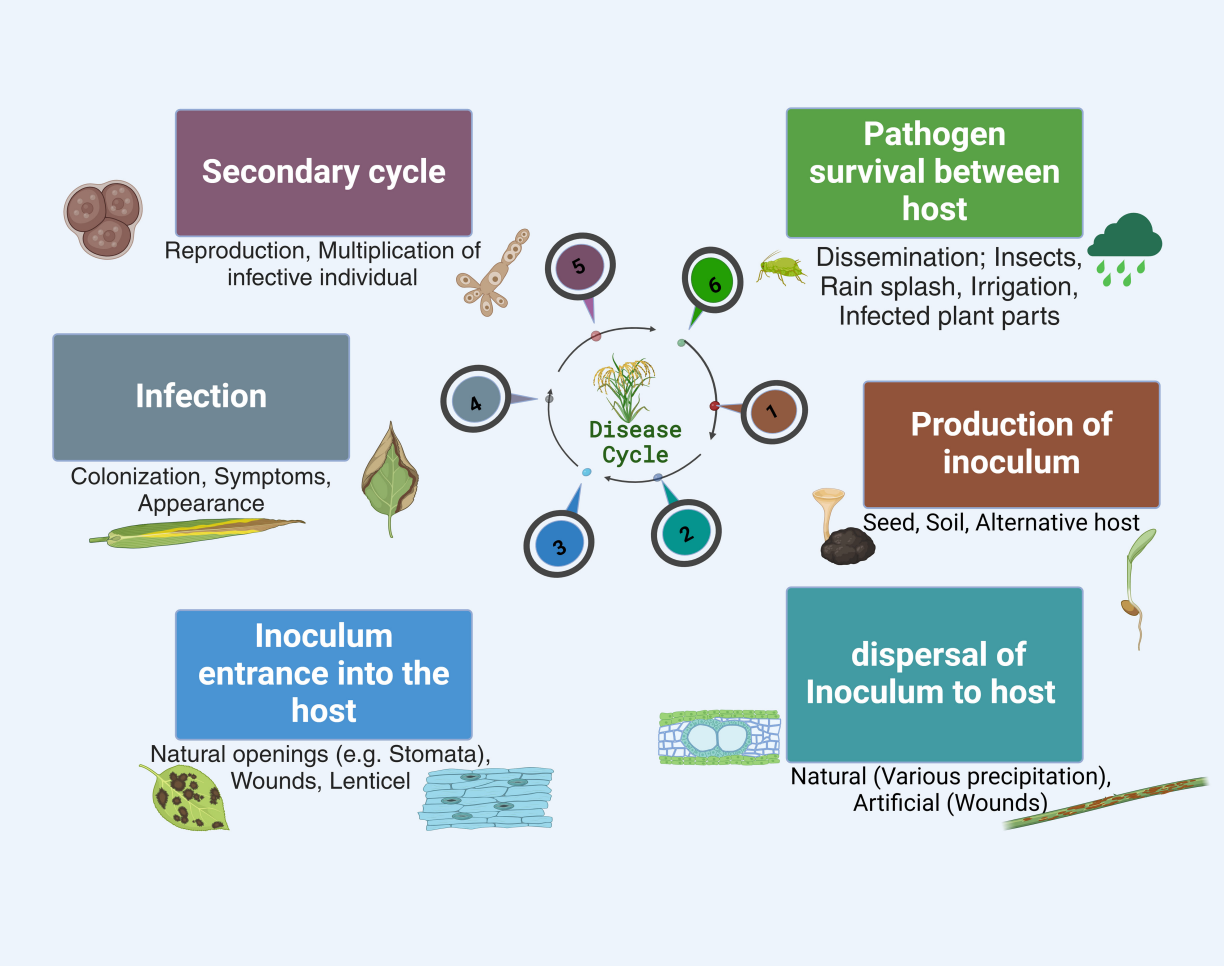
Schematic representation of the plant infection cycle . This shows the plant infection cycle, depicting major stages from the production of inoculum to host colonization and expression of symptoms. The figure highlights interactions between the plant and the pathogen, emphasizing critical checkpoints where the infection process is initiated and possible mitigation of infective individual reproduction until multiplication and dissemination.

Temperature is an important environmental factor influencing the activities of pathogens on plants with a myriad of effects on plant-pathogen interaction (Li et al., 2013). Temperature is the most influencing microclimatic variable in greenhouse experiments in Saudi Arabia (Al-Askar et al., 2022). Temperature influences infection in diverse ways including the rate of pathogenesis and timing of infection (**Figure 1**). Consequently, the medium temperature influences and regulates the rate at which a pathogen will reproduce (Legler et al., 2012). In cases where the timing of infection is important for interactions between two pathogens, the form of competition may be shifted (Al-Naimi et al., 2005), and these changes may affect host susceptibility leading to difficulty in predictions of interactions. A temperature of -13 °C will kill all rust infections (Pfender and Vollmer, 1999), thereby causing a decline in rust survival and overwintering in such weather conditions. Despite this, some spores are suggested to survive better in uredinia (Barua et al., 2018). However, in the broader context of plant pathology, the impact of excessive temperature variations may present a significant obstacle to accurately estimating the frequency and establishment of disease.

High temperature can also confer abiotic stress such as drought stress on the plant by increasing the soil environment temperature which can then affect plant predisposition to pathogen attack (Hunjan and Lore, 2020). Abiotic stress has been observed to contribute to plant susceptibility to pathogens or it may induce general defense pathways that increase resistance (Mittler, 2006). Generally, extreme temperature regimes have been observed to weaken plants’ resistance to disease (Sharath Chandran et al., 2021), and this situation may have influenced the pathogen attack and thwarted the actual measurement of virulence. For example, the virulence of Root-Knot Nematode (RKN) is a measurement of its reproductive factor, and in a situation whereby a plant immunity is weakened as a result of high temperature (Kim et al., 2022), then the RKN reproduction will be unnecessarily high. For this reason, it is noted that at 31°C, more juvenile RKN will penetrate the plants’ root than at 28°C (Yuan et al., 2018). Also, during high temperatures, plants may exhibit stress signs similar to water stress such as wilting, leaf burn, leaf folding, and abscission (Seleiman et al., 2021). Somehow, water stress can also hinder the resistance mechanism of plants (Atkinson and Urwin, 2012). For instance, the resistance mechanism in barley is disrupted when water is restored after drought stress (Guo et al., 2009; Frimpong et al., 2021).

Various studies have indicated that temperature regimes are pathosystem-specific. Moreover, if the greenhouse parameters are not well regulated, a high temperature within the greenhouse can influence the aggressiveness of a pathogen (Chen et al., 2017). Thus, a controllable environment is necessary for studies requiring a particular temperature. Studies have shown that *Globodera pallida* infection on potato plants is optimally favored at a temperature of 15°C (Jones et al., 2017), and rice is better infected by *Xanthomonas oryzae* at a maximum temperature of 35°C and a minimum of 27°C (Horino et al., 1982). High temperature can lead to strain dominance as certain strains that better adapt at higher temperature levels may be more supported (Mariette et al., 2016). New isolates of stripe rust caused by *Puccinia striiformes* f. sp. *Tritici* adapt better than older isolates at a higher temperature regime (Milus et al., 2009). Altogether, this indicates that experiments done in the hot greenhouse in a climate like that of Saudi Arabia can favor the establishment of a high-temperature inclined strain over others. Spores numbers and lesion size are amplified more under conditions of higher temperatures. In some cases of extreme or high temperatures, the establishment of fungal pathogens can be impeded and this can influence the outcome of research requiring the quantification of spores and lesion size. In a study involving *Phakopsora pachyrhizi* infection on soybean, urediniospores failed to develop at a temperature above 37 °C, likewise, no traces of lesion were observed (Bonde et al., 2012). This implies that disease incidence is delayed in the months when the temperature peaks. Greenhouses in Saudi Arabia feature this condition between June and August. All these are indices pointing to the importance of controllable greenhouse parameters in the arid region.

Plant responses to external *stimuli* are spontaneous under a higher temperature regime (Obrepalska-Steplowska et al., 2015). A study by Zhang and co (Zhang et al., 2012) shows that the turnip crinkle virus (TCV) will replicate relentlessly under high temperatures. Similarly, the incidence of leaf blight of onion caused by *Xanthomonas campestris* will be relatively high during July and August when the temperature will be at its peak (Schwartz et al., 2003). This indicates that the bacterium infection is highly favored under high-temperature conditions. However, when the temperature continues to increase, the intensity of the blight begins to reduce because temperatures above 35°C are generally not favorable for the disease (Schwartz et al., 2003). We now have to imagine how the bacteria will behave on onions under temperatures conditions higher than 35°C which is prevalent during the summer months of July and August in Saudi Arabia. It may be difficult to get a proper understanding of the bacteria-onion interaction during these months. The implication is that the execution of experiments will be limited to a certain period of the year except if greenhouses are specially constructed and equipped such that the weather parameters are adequately controlled, and achieving this requires spending more on greenhouse technology.

Additionally, temperature can directly affect host plants genetically (Sbeiti et al., 2023). For example, the resistance conferred on tomato plants carrying the *Mi*-1 gene to *Meloidogyne incognita* is lost at a soil temperature above 32°C (Abdul-Baki et al., 1996; Devran and Özalp, 2018). *M. incognita* reproduced abundantly on the resistant variety exposed to high soil temperature, while the resistance persisted at a lower soil temperature. Moreover, the nematode sex allotment is influenced by temperature, therefore, high temperature will favor the determination of more nematode males which are not of much concern in the infection process (Papadopoulou and Traintaphyllou, 1982). This will hamper the reproduction cycle and overall infection process.

Experimental probes to help determine the temperature ranges at which resistance persists are necessary to determine resistance plant varieties under the plant pathogen management option. However, the *Mi*-1 gene effectiveness can be lost at temperatures above 28 °C (Abdul-Baki et al., 1996; Dropkin, 1969). Fortunately, the resistance capacity can be recovered when plants previously exposed to unfavorable high temperatures are subsequently maintained at a low temperature (Marques de Carvalho et al., 2015). Aside from this phenomenon in the pathosystem as a result of extreme temperature, symptom masking is another interesting observation in plant pathosystems.

Notably, there are instances when infections are not observed on plants due to high temperatures in the greenhouse, especially in plant-virus interactions. Studies based on of tobacco mosaic virus of *Nicotiana benthamiana* have demonstrated this. At 35°C, chlorosis appears instead of the typical necrotic local lesion that is typically associated with the disease (Szittya et al., 2003). Similarly, at higher temperatures, the wilting of tomatoes infected with *Fusarium* wilt was accompanied by leaf yellowing as opposed to lower temperatures when the wilting was absent (Larkin and Fravel, 2002; Thomidis et al., 2023). Conversely, higher temperatures trigger a defense response in plants infected with viruses leading to fewer symptoms due to RNA silencing (Chellappan et al., 2005). It is common to encounter the heat masking phenomenon whereby symptoms of infection especially in virus-plant interaction are lessened under high temperatures (Kassanis, 1957; Ghoshal and Sanfaçon, 2015; Tsai et al., 2022). It is this observation that informs the use of thermotherapy as a physical method of reducing the viral load of planting material (Manganaris et al., 2003). The symptoms of potato leaf roll on *Physalis floridana* fluctuate greatly at varying temperatures. The virus transit within the plant is highly affected by temperature which can lead to differential sampling results especially when only the inoculated leaves are sampled (Kassanis, 1957). Temperature also has a mark on the treatment aspect of an experiment such as fungicide. The effectiveness and duration of efficacy of fungicides are affected by increased temperature (Juroszek et al., 2022). Taken together, the temperature is considered an important factor in plant-pathogen interaction, and conditions leading to its fluctuations should be considerably managed. Despite all the important roles temperature has displayed in various pathosystems inside the greenhouse, the greenhouse moisture has carried an almost similar weight.

### 5.2 Role of humidity in disease proliferation

Disease prevalence is considerably influenced by moisture content. In the greenhouse, moisture supplies are mainly through irrigation to the surface of the plant and root environment, and evaporative cooling, if any. Moisture can affect plant-pathogen interaction in several ways. For instance, fungal spore growth is highly favored by moisture, promoting germ tube elongation, and overall host penetration (Roger et al., 1999). Similarly, bacteria and nematodes require moisture for various survival activities, such as motility and spread, on their host (Fujimoto et al., 2010). Likewise, certain bacteria, either through artificial inoculation or natural infection, require a substantial amount of humidity to facilitate a successful establishment. Implicatively, a moist environment is required for establishing a bacterial pathosystem. It is recommended that bacteria should get a film of water that is at least bigger than the bacteria’s size during proliferation on the plant surface. Primarily, the film provides a necessary aqueous environment that supports bacterial life and facilitates biofilm formation, which is essential for bacterial colonization and host interaction (Grinberg et al., 2019). Notably, Bacteria often face desiccation on plant leaves under fluctuating moisture levels. Moisture availability can influence the resistance mechanism of plants by altering the plant’s immune response and the pathogen’s ability to infect. High humidity inhibits plant immunity by altering the innate signaling pathways (Yao et al., 2023). Likewise, soil moisture levels can modulate microbial communities that directly influence the promotion or suppression of plant disease (Chen et al., 2023). For example, severe leaf symptoms are observed in *Parthenocissus quinquefolia* when infected with *Xylella fastidiosa* (McElrone et al., 2001), and regular watering has been proposed as a control for this disease. These findings highlight the importance of maintaining balanced moisture levels for effective disease management.

The amount of moisture can also directly affect host susceptibility. High moisture levels increase plant succulence, thus, making the plant more vulnerable to infection. However, excessively high moisture levels are often unfavorable for disease development. Gupta et al. (2019) suggested that a medium RH level of about 50% is usually favorable for disease development. For instance, downy mildew development has been shown to increase at maximum humidity (Sun et al., 2017), while onion foliar diseases are more severe at RH above 80% (Tho et al., 2019). Thus, it is apparent from different studies that plant pathogens require a persistent moisture period for development, highlighting the need for precise RH regulation in greenhouses. In arid regions, greenhouses may face challenges in providing consistent humidity levels due to the limited availability of water and fluctuating environmental conditions. For instance, Ascochyta blight caused by the fungus *Mycosphaerella pinodes* develops hastily (Kerling, 1949), especially under wet conditions (Roger et al., 1999), which also facilitates the ease of spread of the disease from one plant part to another part, and from plant to plants. Therefore, to optimize greenhouse conditions in arid regions, proper humidity control systems are essential. Essentially, achieving stable RH levels through evaporative cooling and moisture sensors can significantly enhance pathogen management. This approach is particularly recommended for arid climates, where experimental results and crop productivity can be compromised by extreme environmental conditions. In conclusion, by integrating precision technologies into greenhouse systems, consistent humidity and other factors in line with the requirement of specific pathosystems will be achieved, ensuring the generation of reliable data at optimal plant-pathogen relationship.

## 6 Strategies for controlling greenhouse microclimates in hot arid regions

Greenhouses provide controlled environmental conditions essential for optimizing crop performance, especially in hot arid regions where external conditions are extreme. The efficiency of energy transfer and water vapor flux within a greenhouse depends on its design, cladding materials, shape and orientation, and microclimate control equipment (El Afou et al., 2015). Ensuring a proper greenhouse design and management is crucial to achieving favorable growing conditions and mitigating the unique challenges of arid climates. In arid regions with characteristics of high temperature and low humidity, effective cooling and ventilation systems are crucial. Natural ventilation has been a traditional method utilized in greenhouse cooling but it is most preferred in mild-climate countries and less effective under extreme heat (del Sagrado et al., 2016). In addressing this, advanced cooling techniques such as evaporative cooling and shading systems have been suggested as a suitable integration into the greenhouse structure. Additionally, constructing greenhouses with materials that can reflect solar radiation and that can retain thermal insulation can significantly reduce internal temperatures (Berroug et al., 2011). Primarily, energy and water efficiency are central to greenhouse operations in arid environments. Remarkably, automated and intelligent control systems that monitor and regulate temperature, humidity, and lighting in real-time are available to optimize resource use and operational cost (Çaylı et al., 2017; Zhang et al., 2020). These systems provide relevant precision in managing microclimates and ensuring that crops receive the optimal conditions for growth. By adopting these strategies, greenhouses in hot arid regions can achieve the balance required for effective conditions for an optimal crop production environment. There are nevertheless several ways to achieve this. For example, the appropriate greenhouse construction pattern for cooling is a necessity in hot arid regions.

### 6.1 Greenhouse design and orientation

Effective greenhouse design and orientation are critical for optimizing temperature and humidity control and minimizing energy use in hot arid regions. Remarkably, research has shown that the orientation of the greenhouse can be manipulated to control the environment inside the greenhouse (Dragicevic, 2011). Greenhouse orientation determines the exposure to solar radiation, which directly impacts internal temperatures. Research highlights that an East-West (E-W) orientation is ideal for reducing solar gain in high-temperature areas, such as Saudi Arabia, particularly at latitudes like 24 °N. This orientation minimizes summer radiation while maximizing winter sunlight, as confirmed by studies across various latitudes (Dragicevic, 2011; El-Maghlany et al., 2015). However, regions around the equator may require alternative alignment that adapts to their unique solar pattern, since it receives more solar radiation in the winter and less in the summer.

In addition to orientation, the shape of the greenhouse also influences its thermal efficiency. This greenhouse feature has offered a great effect in controlling greenhouse temperature (Choab et al., 2019). Many studies on the preferred shapes and forms of greenhouse have demonstrated that the uneven-span-shaped greenhouse can receive maximum radiation (Soussi et al., 2022). Minimum solar radiation has been demonstrated to be received by the Quonset and vinery-shaped greenhouse (Mobtaker et al., 2019). On considering the heating requirement of some common greenhouse shapes, Gupta and Chandra (2002) found that the Quonset shapes require the most heating due to their capacity for minimized radiation collection. The Quonset shape generally presents the least temperature and solar collection, unlike the uneven-span shape (Sethi, 2009; Choab et al., 2019). The single-span Quonset shape is predominantly used in the Arabian Peninsula (Al-Mulla, 2006), and the combination with the most appropriate covering material is most suitable for cooling greenhouses in arid regions.

### 6.2 Covering materials for greenhouses

The choice of covering material significantly influences the greenhouse microclimate, particularly in hot arid environments. Attempts have been made to identify the most suitable greenhouse cover by studying the properties of some covering materials as well as enhancing existing material with additional substances. Abdel-Ghany et al. (2012) highlighted the important requirements greenhouse cover under tough environments should complement. Ideal coverings should support a homogenous distribution of light and allow optimal light penetration while blocking excess heat and near-infrared radiation (NIR). Notably, glass covering is suggested to excel at transmitting photosynthetically active radiation (PAR) which is essential for plant growth (Kittas et al., 1999; Maraveas, 2019). However, despite the effectiveness of the glass cover, some plastic films and sheets possess favorable cover features, such as better NIR reflection, providing a conducive environment for plant activities (García-Alonso et al., 2007). As a result, it can be incorporated into the cooling system of greenhouses in hot, arid regions.

Advanced materials, such as fiberglass and polycarbonate sheets, are heat-resistant and durable (Briassoulis et al., 1997), making them suitable for regions like Saudi Arabia, where extreme temperatures and frequent sandstorms accelerate material degradation. Moreover, fiberglass and polycarbonate plastic sheets are strong, heat-resistant, and excellent at transmitting UV and PAR radiation (Al-Mahdouri et al., 2014). Fiberglass greenhouse supports more evaporative cooling and provides the desired environmental temperature for crops.

**Figure 2:**
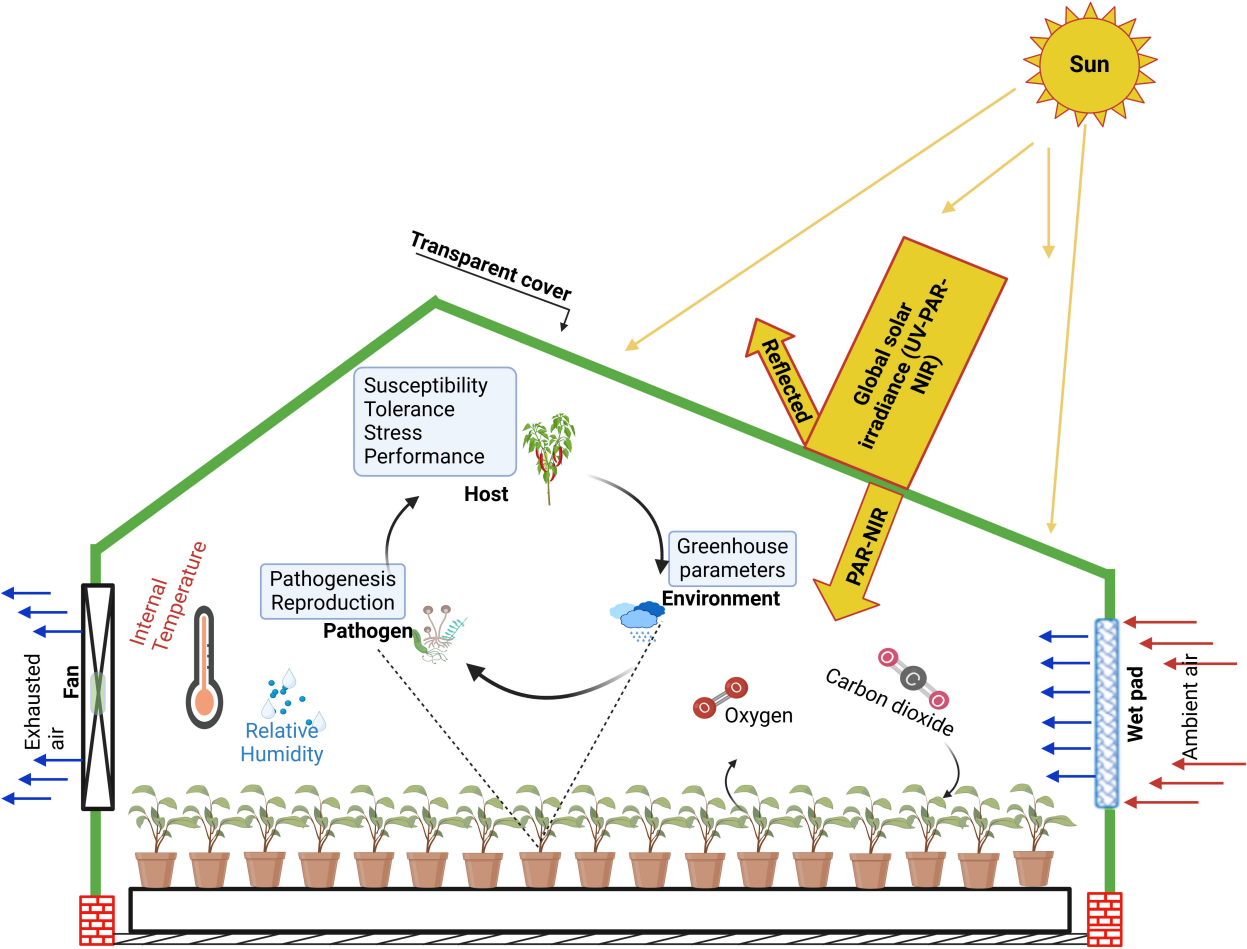
Enhancing greenhouse microclimate with wet-pad and fans cooling system: A schematic guide to plant-pathogen interactions. This demonstrates the impact of a cooling system on mitigating extreme environmental conditions. the figure links improved microclimatic regulation to stress regulation on crops and suggests the modulation of plant-pathogen interaction, providing a conceptual framework for optimizing crop health in a protected and controlled environment. The greenhouse cover selectively filters solar radiation, to retain near-infrared radiation (NIR) and photosynthetically active radiation (PAR), which are essential for plant growth. This integrated approach to greenhouse technology in extreme arid conditions depicts the ultimate promotion of optimal environments required by crops and interactions with pathogens.

Additionally, innovative solutions, such as liquid-filled roofs and reflective solid covers, are observed to further enhance cooling by absorbing or reflecting solar radiation. However, these systems can be resource-intensive, requiring significant water or material inputs (Abdel-Ghany et al., 2001). Similarly, biodegradable paper (Briassoulis and Giannoulis, 2018), shade clothes, and even brick walls (Zhang et al., 2017) have been adopted as covering materials for greenhouses in some regions. Altogether, an approach combining ventilation, evaporative cooling, and NIR-reflective films offers a practical solution for managing heat load while maintaining crop health (**Figure 2**).

In addition to the greenhouse design and choice of cover material, important factors to consider during installation are the size of the greenhouse and the capacity of the cooling system (Dougka and Briassoulis, 2020). This consideration is a huge determinant of the efficiency and durability of the whole greenhouse system. To attain a suitable microclimate, the cooling system size to greenhouse size ratio should be appropriated. This is because excessive heat can build up when the cooling system provides less than what the greenhouse capacity requires.

Consequently, the necessity to provide a comfortable environment for crops inside greenhouses in hot climates has prompted the development of various cooling technologies that involve the replacement of the hot air present inside the greenhouse with the cooler air outside, through methods such as heat exchangers and evaporative cooling.

### 6.3 Advanced cooling systems and technologies

In mild climates, cooling the greenhouse can be a simple technique that regulates extremes of humidity and temperature through the management of natural ventilation (Abreu and Meneses, 1994). However, using just ventilation as a cooling strategy can be appropriate when there is a low outside temperature and a moderate inside temperature. In arid climates, where ambient temperatures often exceed 45 °C, cooling systems should be very efficient and sustainable. Besides, ventilative cooling in hot arid regions is hard to achieve due to the low relative humidity during summer in arid regions. Therefore, optional methods such as evaporative cooling can substitute ventilation in hot regions. Greenhouse temperature under evaporative cooling can be up to 12°C below the ambient level (Landsberg et al., 1979). Evaporative cooling is more efficient in hot climates because of the presence of dryer air which produces better cooling results than wet air. The principle of heat and mass exchange between air and water in the wet pad is utilized in evaporative cooling systems (Arbel et al., 1999; Patel and Sheth, 2015). When water that is exposed to evaporation moves into air space, the air temperature is reduced through the conversion of sensible heat to latent heat. Evaporative cooling systems can be the fan and pad system, fogging system, and roof evaporation cooling technique (von Zabeltitz, 2011; Soussi et al., 2022), the use of any of which will reduce the ambient temperature of greenhouses to a level suitable for great crop performance.

The fogging system entails pumping water through a fogging nozzle from above the greenhouse. This process humidifies the greenhouse’s air, bringing about the cooling effect (Montero et al., 1990). The system is cost-effective, ensuring uniform humidity and air temperature distribution (Abdel-Ghany and Kozai, 2006). Fogging efficiency depends on system design, such as high-pressure nozzles, 40 bars, producing 10-30 µm droplets, or low-pressure ones, (5 bars, producing 200 µm) (Kittas et al., 2012). Despite its benefits, the fogging system is water intensive and relies on fresh water, making it less suitable for arid regions like the Arabian Peninsula. It is thus not highly recommended for greenhouses in arid regions where the availability of fresh water is a huge concern. Additionally, it can lead to excessive water accumulation on plants’ surfaces, which can encourage disease development (Kamel et al., 2008). Also, in situations where less quality water is used, there is the possibility of salt precipitation deposition on the plants (Arbel et al., 1999). These limitations necessitate alternative cooling techniques, such as the fan and pad, which is also a popular greenhouse cooling technique in Saudi Arabia, especially during the summer. The fan and pad technique is considered an efficient greenhouse microclimate control method wherever the outside temperature is above 40°C (Soussi et al., 2022). In the simplest setup, it consists of a wet pad on one side of the greenhouse and electrically propelled fans on the opposite side, driving airflow across the pad. Cooling efficiency depends on pad material, thickness, and airflow rate (Liao and Chiu, 2002). Common pad materials include the aspen pad, plastic fiber, palm dates, and corrugated cellulose (Al-Helal, 2009). The appropriate size of the cooling pad depends on the type of pad material. It is recommended that the length of the pad should correspond to the length of the wall (Park et al., 2022). For cellulose material, an airflow of around 70 cubic meters per minute is required for each square meter of pad (Watson et al., 2019). Optimizing pad size and airflow is crucial for achieving desired cooling levels. For instance, Kittas et al. (2012) recommended 1 m^2^ of pad area per 20 – 30 m^2^ of the greenhouse, with an air flow of 120 to 150 m^3^/hour. For a standard 10 cm thick corrugated cellulose pad, a face velocity of 1.27 m/s is recommended by the American Society of Agricultural Engineering (ASAE, 2000). The outcome of the work of Al-Helal and Al-Toweejre. (2001) showed that the corrugated cellulose pad of 10 cm thickness provided a higher cooling efficiency than the same padding material but of 5 cm thickness. Remarkably, the system can lower greenhouse temperature by 4°C below the ambient temperature.

The pad becomes clogged due to salt accumulation during evaporation and the impact of accumulated dust from the surroundings. Therefore, maintenance and possible replacement costs may occur when the strength, water absorption capacity, and other physical properties have depreciated.

However, in arid regions, high salinity of impure water can lead to pad clogging due to salt accumulation during evaporation, reducing cooling efficiency and increasing energy consumption (Al-Helal, 2007). Over time, pad clogging and dust accumulation will necessitate frequent maintenance, and possible replacement may occur when the strength, water absorption capacity, and other physical properties have depreciated.

Evaporative cooling systems remain effective in arid conditions but require supplementation with shading techniques to mitigate excessive heating. Shading reduces solar radiation and heat load, addressing the high temperature of peak summer months (Abad et al., 2007; Ali and Albayati, 2017). Such strategies are crucial for optimizing greenhouse operations in hot arid regions of the Arabian Peninsula.

### 6.4 Shading techniques to enhance cooling efficiency

Shading is a system embrassed in greenhouses to maintain a suitable temperature by reducing transmitted solar radiation (Ahmed et al., 2019). The ideal greenhouse cover should be able to filter out unnecessary spectra components, allowing only the required light for plant growth. Plants require PAR for growth, while NIR contributes primarily to heat, making it less beneficial to the plant (Kempkes et al., 2012). Effective shading techniques aim to reduce NIR transmission to prevent overheating. Fortunately, UV-absorbing plastic films are widely available to filter UV radiation, leaving only the NIR as the primary source of unnecessary heat (Kempkes et al., 2009).

In arid regions, shading is crucial in reducing solar radiation load during peak temperatures (Abdel-Ghany et al., 2015; Hirich and Choukr-Allah, 2017; Fatnassi et al., 2023). This approach not only protects plants but also mitigates the rapid degradation of greenhouse covers as a result of the harsh climate (Abdel-Ghany et al., 2012). Traditional shading techniques include spraying an aqueous solution of hydrated calcium oxide on greenhouse exteriors (Mashonjowa et al., 2010), which can lower temperatures by up to 10°C (Omar et al., 2021). However, this method can have a mitigating effect on plant growth because it reduces both PAR and non-PAR radiation (Baille et al., 2001). Additionally, the method requires frequent application of the solution because its durability is limited especially during rainfall (Abdel-Ghany et al., 2012).

Other conventional shading methods include the use of fixed or movable curtains and plastic nets of various types and colors (Abdel-Ghany et al., 2015). Studies have shown a preference for white plastic nets for optimal crop performance (Medany et al., 2009). Typically, the mesh size is often considered during selection as it influences shading efficiency and microclimate regulation. Harmanto et al. (2006) recommended A mesh size of 52 due to its natable temperature control and pest prevention.

Depending on the desired effect, shading can be applied internally or externally. External shading is popular for heat reduction and improved ventilation. Abdel-Ghany et al. (2015) have confirmed that thermal reduction of up to 16°C in the greenhouse is possible with external shading.

Additionally, the available ventilation is another consideration when determining shading technique. Internal shading, although effective in maintaining temperature during the cooler season, can disturb natural ventilation and affect microclimate balance.

Similarly, different shading material color gives different crop growth levels (Mascarini et al., 2001). Thus, choosing the color that supports optimal efficiency is very crucial. For instance, white shading clothes at 40% shade effectively reduces the air temperature for optimal crop growth (Willits, 2001). Likewise, Tang et al. (2020) found that shading material influences light regulation and can enhance yield by up to 1.2 times compared to unshaded ones. Overall, Greenhouse technology continues to evolve, with ongoing advancement promising more efficient and sustainable solutions to optimize greenhouse microclimatic conditions for all greenhouse activities.

## 7 Future trends and innovations

Greenhouse technology has witnessed tremendous improvement over the years and research to enhance the technology is ongoing. Greenhouse technology that initially focused on the distribution and design of lighting and ventilation structure types has now developed into the installation of automation systems and sensors (Aznar-Sánchez et al., 2020). The differences in external climatic conditions of greenhouses in different climatic areas are a reason why greenhouses face different challenges, and the automation systems put in place within them need to be modified to take into account these particular environmental aspects. For example, to enable optimal plant growth in colder places, greenhouse automation systems need to concentrate on effective heating mechanisms, insulation, and temperature management. In warmer climes, on the other hand, the focus can be on efficient cooling systems, shade management, and ventilation to avoid overheating and preserve a suitable habitat for plants, and other systems intended.

The challenging season for greenhouse cultivation in the desert of arid regions is the summer where high temperature and low humidity are the limiting factors. Currently, a popular corrective measure is the use of evaporative cooling which consequently has some issues with water use. However, recent advancements in greenhouse technologies propose suggestions to address issues associated with regulating extreme temperatures in desert region greenhouses. New greenhouse covering materials are being developed such that the optic properties of the material can be actively switchable. The work of Baeza et al. (2020) demonstrated, with the aid of simulation tools, the advantages switchable properties have over permanent properties where they were able to show considerable water saving for the pad system when a switchable cover was used. In the hot regions where available heat in the daytime is much, this can be exploited to provide a balanced heat requirement for the greenhouse through the day and night. It is suggested that the heat generated in the daytime can be stored in mediums that will release the stored heat at nighttime to warm up the greenhouse in winter (Soussi et al., 2022).

Knowledge-based methods for plant-pathogen-environment interaction studies have been deduced from integrating implemented sensors and machine learning into greenhouse technology (Kuska et al., 2022). This approach has presented the potential for quick and active identification of necessary parameters during plant-pathogen interaction inside the greenhouse. Intelligent technologies can be used to predict the greenhouse microclimate which can be translated into information necessary to maintain the required greenhouse conditions (Taki et al., 2018; Sedig et al., 2019). A convex bidirectional extreme learning machine (CB-ELM) has been developed to predict greenhouse temperature and humidity (Zou et al., 2017).

Additionally, recommendations have been made on manipulating the resources unique to the Arabian Peninsula such as intense sunlight and saline sea water into emerging technologies for advanced greenhouse technology concepts. An interesting outlook is the probable use of transparent infrared harvesting solar cells that will allow low-energy cooling, temperature reduction, and even energy generation (Lefers et al., 2020). This effort will lead to reduced freshwater requirement; which is of primary consideration in the Arabian Peninsula. With this concept, there is hope for greenhouses that are energy efficient in the provision of a controlled environment in the Arabian Peninsula.

## 8 Conclusion

Greenhouse technology plays a great role in modern agriculture, particularly in regions with extreme climatic conditions, by offering a controlled environment for continuous crop production and detailed study of plant-pathogen interactions. The success of greenhouse cultivation especially for plant pathology research in hot arid regions lies in the ability to maintain optimal microclimatic conditions that support plant health while effectively managing pests and diseases.

This review has highlighted the critical need for innovative cooling strategies in greenhouses to ameliorate the harsh summer conditions of the Arabian peninsula. Through an extensive examination of various cooling techniques, principles, and effectiveness, we have identified promising approaches that can create a controlled environment conducive to plant, pathogen control, and plant pathology studies. The integration and manipulation of these techniques offer the potential for maintaining a balanced microclimate with minimal resource consumption, thereby ensuring sustainable and efficient greenhouse operations.

In conclusion, the interplay between greenhouse microclimates and plant pathology is crucial for advancing agricultural productivity. Future research and technological advancement should focus on refining these cooling systems to enhance greenhouse functions that optimize plant pathology research output. By doing so, we can ensure that greenhouses offer a viable and effective solution to the challenges facing plant pathology research in particular and crop production in generation in the hot arid region.
